# Poly[[bis­[μ_2_-8-ethyl-5-oxo-2-(piperazin-1-yl)-5,8-dihydro­pyrido[2,3-*d*]pyrimidine-6-carboxyl­ato]manganese(II)] dihydrate]

**DOI:** 10.1107/S1600536808006533

**Published:** 2008-03-14

**Authors:** Jing Huang, Wei-Ping Hu, Zhe An

**Affiliations:** a School of Pharmaceutical Science, Harbin Medical University, Harbin 150086, People’s Republic of China; b Second Hospital, Harbin Medical University, Harbin 150086, People’s Republic of China

## Abstract

In the title compound, {[Mn(C_14_H_16_N_5_O_3_)_2_]·2H_2_O}_*n*_, the Mn^II^ atom (site symmetry 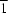) exhibits a distorted *trans*-MnN_2_O_4_ octa­hedral geometry defined by two monodentate *N*-bonded and two bidentate *O,O*′-bonded 8-ethyl-5-oxo-2-(piperazin-1-yl)-5,8-dihydro­pyrido[2,3-*d*]pyrimidine-6-carboxyl­ate anions. An N—H⋯O hydrogen bond is present in the crystal structure. The extended two-dimensional structure is a square grid and the disordered uncoordinated water mol­ecules occupy cavities within the grid.

## Related literature

For background, see: Mizuki *et al.* (1996 ▶).
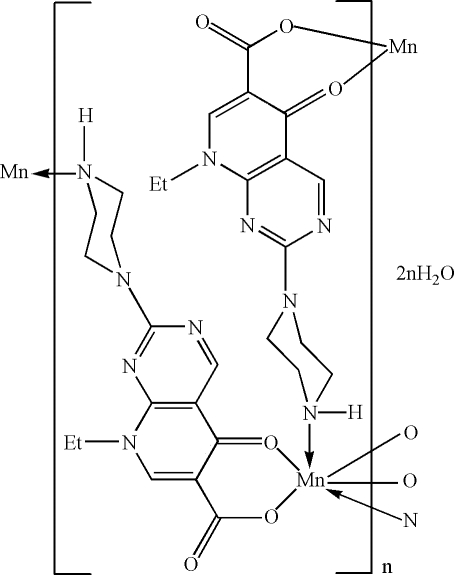


## Experimental

### 

#### Crystal data

[Mn(C_14_H_16_N_5_O_3_)_2_]·2H_2_O
*M* *_r_* = 695.58
Monoclinic, 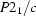

*a* = 6.0422 (2) Å
*b* = 21.5673 (8) Å
*c* = 12.7395 (5) Å
β = 99.617 (1)°
*V* = 1636.8 (1) Å^3^
*Z* = 2
Mo *K*α radiation
μ = 0.47 mm^−1^
*T* = 295 (2) K
0.34 × 0.26 × 0.18 mm

#### Data collection

Bruker SMART CCD diffractometer
Absorption correction: multi-scan (*SADABS*; Bruker, 1998 ▶) *T*_min_ = 0.861, *T*_max_ = 0.910
10030 measured reflections
3938 independent reflections
3465 reflections with *I* > 2σ(*I*)
*R*_int_ = 0.019

#### Refinement

*R*[*F* ^2^ > 2σ(*F* ^2^)] = 0.060
*wR*(*F* ^2^) = 0.191
*S* = 1.10
3938 reflections
227 parameters
1 restraint
H atoms treated by a mixture of independent and constrained refinement
Δρ_max_ = 0.97 e Å^−3^
Δρ_min_ = −0.48 e Å^−3^

### 

Data collection: *SMART* (Bruker, 1998 ▶); cell refinement: *SAINT-Plus* (Bruker, 1998 ▶); data reduction: *SAINT-Plus*; program(s) used to solve structure: *SHELXS97* (Sheldrick, 2008 ▶); program(s) used to refine structure: *SHELXL97* (Sheldrick, 2008 ▶); molecular graphics: *SHELXTL* (Sheldrick, 2008 ▶); software used to prepare material for publication: *SHELXTL*.

## Supplementary Material

Crystal structure: contains datablocks I, global. DOI: 10.1107/S1600536808006533/hb2703sup1.cif

Structure factors: contains datablocks I. DOI: 10.1107/S1600536808006533/hb2703Isup2.hkl

Additional supplementary materials:  crystallographic information; 3D view; checkCIF report

## Figures and Tables

**Table 1:** Selected bond lengths (Å)

Mn1—O1	2.106 (2)
Mn1—O3	2.1667 (16)
Mn1—N5^i^	2.372 (2)

Symmetry code: (i) 
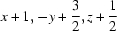
.

**Table 2:** Hydrogen-bond geometry (Å, °)

*D*—H⋯*A*	*D*—H	H⋯*A*	*D*⋯*A*	*D*—H⋯*A*
N5—H5*N*⋯O2^ii^	0.893 (10)	2.268 (12)	3.149 (3)	169 (3)

Symmetry code: (ii) 
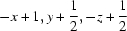
.

**Fig. 1.**
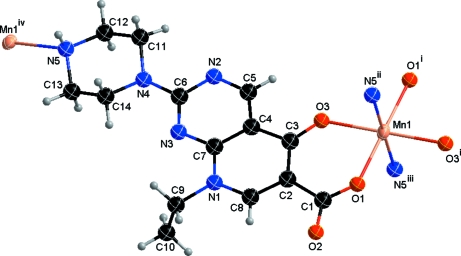
The asymmetric unit of (I) showing the showing 50% displacement ellipsoids (water molecule O atoms have been omitted for clarity).

**Fig. 2.**
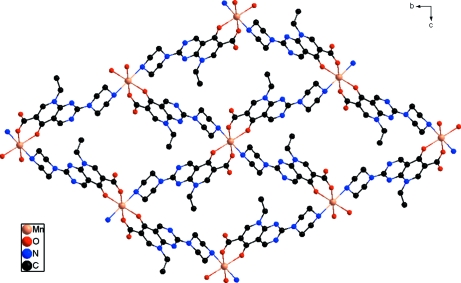
A view of part of a two-dimensional polymeric sheet in (I) showing the square-grid connectivity (H atoms and water molecule O atoms omitted for clarity).

## supplementary crystallographic information

### Comment

Pipemidic acid (Hppa, C_14_H_17_N_5_O_3_, 8-Ethyl-5,8-dihydro-5-oxo-2- (1-piperazinyl)-pyrido(2,3 - d)-pyrimidine-6-carboxylic acid) is member of a class of quinolones used to treat infections (Mizuki *et al.*, 1996). The metal complexes of the ppa anion have not been reported; the title manganese(II) complex, (I), is reported here (Fig. 1).

The Mn^II^ atom in (I) with site symmetry 1 is coordinated by four oxygen atoms and two N atoms from four ppa ligands (two monodentate-N and two O,*O*-bidentate) (Table 1) to form a square grid propagating in (Fig. 2). An N—H···O hydrogen bond (Table 2) helps to stabilize this arrangement.

The disordered, uncoordinated, water molecules occupy cavities within the grid. In the present study, their attached H atoms could not be located.

### Experimental

A mixture of Mn(CH_3_COO)_2_.4H_2_O (0.061 g, 0.25 mmol), Hppa (0.15 g, 0.5 mmol), sodium hydroxide (0.04 g, 1 mmol) and water (12 ml) was stirred for 30 min in air. The mixture was then transferred to a 23 ml Teflon-lined hydrothermal bomb. The bomb was kept at 433 K for 72 h under autogenous pressure. Upon cooling, colourless prisms of (I) were obtained from the reaction mixture.

### Refinement

The carbon-bound H atoms were positioned geometrically (C—H = 0.93–0.97 Å) and refined as riding with *U*_iso_(H) = 1.2*U*_eq_(C). The N-bound H atom was located in a difference map and its position was freely refined with *U*_iso_(H) = 1.2*U*_eq_(N).

The water H atoms could not be placed due to disorder.

### Crystal data

**Table:**

[Mn(C_14_H_16_N_5_O_3_)_2_]·2H_2_O	*F*_000_ = 718.0
*M**_r_* = 695.58	*D*_x_ = 1.399 Mg m^−^^3^
Monoclinic, *P*2_1_/*c*	Mo *K*α radiation λ = 0.71073 Å
Hall symbol: -P 2ybc	Cell parameters from 4747 reflections
*a* = 6.0422 (2) Å	θ = 2.5–28.3º
*b* = 21.5673 (8) Å	µ = 0.47 mm^−^^1^
*c* = 12.7395 (5) Å	*T* = 295 (2) K
β = 99.617 (1)º	Prism, colorless
*V* = 1636.8 (1) Å^3^	0.34 × 0.26 × 0.18 mm
*Z* = 2	

### Data collection

**Table:**

Bruker SMART CCD diffractometer	3938 independent reflections
Radiation source: fine-focus sealed tube	3465 reflections with *I* > 2σ(*I*)
Monochromator: graphite	*R*_int_ = 0.019
*T* = 295(2) K	θ_max_ = 28.3º
ω scans	θ_min_ = 2.5º
Absorption correction: multi-scan(SADABS; Sheldrick, 1996)	*h* = −6→8
*T*_min_ = 0.861, *T*_max_ = 0.910	*k* = −27→28
10030 measured reflections	*l* = −15→16

### Refinement

**Table:**

Refinement on *F*^2^	Secondary atom site location: difference Fourier map
Least-squares matrix: full	Hydrogen site location: inferred from neighbouring sites
*R*[*F*^2^ > 2σ(*F*^2^)] = 0.061	H atoms treated by a mixture of independent and constrained refinement
*wR*(*F*^2^) = 0.191	*w* = 1/[σ^2^(*F*_o_^2^) + (0.1066*P*)^2^ + 1.0983*P*] where *P* = (*F*_o_^2^ + 2*F*_c_^2^)/3
*S* = 1.11	(Δ/σ)_max_ < 0.001
3938 reflections	Δρ_max_ = 0.98 e Å^−^^3^
227 parameters	Δρ_min_ = −0.48 e Å^−^^3^
1 restraint	Extinction correction: none
Primary atom site location: structure-invariant direct methods	

### Special details

**Table:**

Geometry. All e.s.d.'s (except the e.s.d. in the dihedral angle between two l.s. planes) are estimated using the full covariance matrix. The cell e.s.d.'s are taken into account individually in the estimation of e.s.d.'s in distances, angles and torsion angles; correlations between e.s.d.'s in cell parameters are only used when they are defined by crystal symmetry. An approximate (isotropic) treatment of cell e.s.d.'s is used for estimating e.s.d.'s involving l.s. planes.
Refinement. Refinement of *F*^2^ against ALL reflections. The weighted *R*-factor *wR* and goodness of fit *S* are based on *F*^2^, conventional *R*-factors *R* are based on *F*, with *F* set to zero for negative *F*^2^. The threshold expression of *F*^2^ > σ(*F*^2^) is used only for calculating *R*-factors(gt) *etc*. and is not relevant to the choice of reflections for refinement. *R*-factors based on *F*^2^ are statistically about twice as large as those based on *F*, and *R*- factors based on ALL data will be even larger.

### Fractional atomic coordinates and isotropic or equivalent isotropic displacement parameters (Å^2^)

**Table:**

	*x*	*y*	*z*	*U*_iso_*/*U*_eq_	Occ. (<1)
Mn1	0.5000	0.5000	0.5000	0.02831 (19)	
O1W	0.388 (4)	0.5235 (7)	0.9613 (10)	0.244 (9)	0.50
O1	0.7068 (3)	0.49893 (7)	0.38228 (16)	0.0341 (4)	
O2W	−0.081 (2)	0.4426 (8)	0.9246 (7)	0.194 (6)	0.50
O2	0.8721 (5)	0.51863 (13)	0.2451 (2)	0.0717 (8)	
O3	0.3574 (3)	0.58106 (8)	0.41354 (14)	0.0378 (4)	
N1	0.5078 (5)	0.67158 (12)	0.1504 (2)	0.0533 (7)	
N2	−0.0007 (4)	0.73723 (11)	0.2985 (2)	0.0464 (6)	
N3	0.2395 (4)	0.74681 (10)	0.16596 (18)	0.0432 (5)	
N4	−0.0102 (4)	0.82368 (10)	0.19027 (19)	0.0384 (5)	
N5	−0.2339 (3)	0.93726 (9)	0.11005 (17)	0.0322 (4)	
H5N	−0.140 (4)	0.9649 (12)	0.146 (2)	0.048*	
C1	0.7282 (4)	0.52991 (11)	0.3018 (2)	0.0358 (5)	
C2	0.5782 (4)	0.58537 (11)	0.2731 (2)	0.0354 (5)	
C3	0.4044 (4)	0.60559 (10)	0.33118 (19)	0.0313 (5)	
C4	0.2852 (4)	0.66087 (11)	0.28824 (19)	0.0336 (5)	
C5	0.1042 (5)	0.68542 (12)	0.3304 (2)	0.0425 (6)	
H5A	0.0546	0.6635	0.3848	0.051*	
C6	0.0805 (4)	0.76813 (12)	0.2189 (2)	0.0360 (5)	
C7	0.3383 (5)	0.69362 (12)	0.2010 (2)	0.0395 (6)	
C8	0.6170 (5)	0.61896 (14)	0.1875 (2)	0.0496 (7)	
H8A	0.7282	0.6046	0.1512	0.059*	
C9	0.5717 (8)	0.7043 (2)	0.0566 (3)	0.0725 (12)	
H9A	0.5531	0.7486	0.0645	0.087*	
H9B	0.7285	0.6963	0.0537	0.087*	
C10	0.4373 (10)	0.6840 (4)	−0.0400 (5)	0.116 (2)	
H10A	0.4583	0.6403	−0.0488	0.174*	
H10B	0.4811	0.7059	−0.0989	0.174*	
H10C	0.2822	0.6923	−0.0375	0.174*	
C11	−0.1446 (6)	0.85820 (14)	0.2560 (2)	0.0499 (7)	
H11A	−0.0477	0.8852	0.3045	0.060*	
H11B	−0.2170	0.8295	0.2980	0.060*	
C12	−0.3226 (5)	0.89702 (13)	0.1855 (2)	0.0433 (6)	
H12A	−0.4326	0.8693	0.1460	0.052*	
H12B	−0.3996	0.9225	0.2308	0.052*	
C13	−0.1018 (4)	0.89946 (12)	0.0466 (2)	0.0372 (5)	
H13A	−0.0347	0.9264	−0.0003	0.045*	
H13B	−0.2015	0.8710	0.0025	0.045*	
C14	0.0812 (4)	0.86281 (12)	0.1146 (2)	0.0382 (6)	
H14A	0.1573	0.8371	0.0693	0.046*	
H14B	0.1906	0.8911	0.1530	0.046*	

### Atomic displacement parameters (Å^2^)

**Table:**

	*U*^11^	*U*^22^	*U*^33^	*U*^12^	*U*^13^	*U*^23^
Mn1	0.0350 (3)	0.0187 (3)	0.0304 (3)	−0.00041 (16)	0.0030 (2)	0.00255 (16)
O1W	0.47 (3)	0.155 (12)	0.111 (9)	0.000 (15)	0.058 (14)	−0.057 (9)
O1	0.0407 (10)	0.0237 (9)	0.0383 (10)	0.0041 (6)	0.0073 (8)	0.0040 (6)
O2W	0.203 (11)	0.307 (17)	0.088 (6)	−0.110 (12)	0.076 (7)	−0.028 (8)
O2	0.0854 (18)	0.0730 (16)	0.0674 (16)	0.0491 (15)	0.0441 (14)	0.0338 (13)
O3	0.0448 (10)	0.0297 (9)	0.0403 (9)	0.0085 (7)	0.0110 (7)	0.0122 (7)
N1	0.0730 (17)	0.0433 (13)	0.0500 (14)	0.0245 (12)	0.0294 (12)	0.0196 (11)
N2	0.0498 (13)	0.0386 (12)	0.0550 (14)	0.0152 (10)	0.0211 (11)	0.0213 (11)
N3	0.0549 (13)	0.0339 (11)	0.0434 (12)	0.0165 (10)	0.0157 (10)	0.0150 (9)
N4	0.0418 (11)	0.0301 (10)	0.0458 (12)	0.0107 (9)	0.0146 (9)	0.0133 (9)
N5	0.0344 (10)	0.0231 (9)	0.0382 (10)	0.0029 (7)	0.0034 (8)	0.0012 (8)
C1	0.0410 (13)	0.0311 (12)	0.0355 (12)	0.0080 (10)	0.0071 (10)	0.0029 (9)
C2	0.0429 (13)	0.0281 (11)	0.0360 (12)	0.0084 (9)	0.0092 (10)	0.0040 (9)
C3	0.0367 (11)	0.0227 (10)	0.0337 (11)	0.0032 (9)	0.0033 (9)	0.0042 (8)
C4	0.0396 (12)	0.0265 (11)	0.0352 (12)	0.0060 (9)	0.0073 (9)	0.0067 (9)
C5	0.0491 (15)	0.0339 (13)	0.0475 (15)	0.0103 (11)	0.0167 (12)	0.0168 (11)
C6	0.0377 (12)	0.0307 (12)	0.0398 (13)	0.0066 (9)	0.0066 (10)	0.0090 (10)
C7	0.0506 (14)	0.0313 (12)	0.0386 (13)	0.0109 (11)	0.0132 (11)	0.0082 (10)
C8	0.0609 (17)	0.0438 (15)	0.0485 (16)	0.0220 (13)	0.0219 (13)	0.0120 (12)
C9	0.091 (3)	0.071 (2)	0.065 (2)	0.035 (2)	0.041 (2)	0.0293 (19)
C10	0.111 (4)	0.154 (6)	0.085 (4)	0.032 (4)	0.022 (3)	0.021 (4)
C11	0.0604 (17)	0.0465 (15)	0.0478 (15)	0.0251 (14)	0.0234 (13)	0.0176 (13)
C12	0.0427 (13)	0.0368 (13)	0.0535 (16)	0.0129 (11)	0.0169 (12)	0.0133 (12)
C13	0.0394 (13)	0.0308 (12)	0.0414 (13)	0.0087 (10)	0.0071 (10)	0.0078 (10)
C14	0.0365 (12)	0.0304 (12)	0.0497 (14)	0.0075 (9)	0.0128 (11)	0.0136 (10)

### Geometric parameters (Å, °)

**Table:**

Mn1—O1	2.106 (2)	C2—C8	1.361 (4)
Mn1—O1^i^	2.106 (2)	C2—C3	1.450 (3)
Mn1—O3	2.1667 (16)	C3—C4	1.452 (3)
Mn1—O3^i^	2.1667 (16)	C4—C7	1.399 (3)
Mn1—N5^ii^	2.372 (2)	C4—C5	1.400 (4)
Mn1—N5^iii^	2.3723 (19)	C5—H5A	0.9300
O1—C1	1.248 (3)	C8—H8A	0.9300
O2—C1	1.244 (3)	C9—C10	1.425 (8)
O3—C3	1.249 (3)	C9—H9A	0.9700
N1—C8	1.357 (3)	C9—H9B	0.9700
N1—C7	1.382 (4)	C10—H10A	0.9600
N1—C9	1.493 (4)	C10—H10B	0.9600
N2—C5	1.315 (3)	C10—H10C	0.9600
N2—C6	1.371 (4)	C11—C12	1.531 (4)
N3—C7	1.335 (3)	C11—H11A	0.9700
N3—C6	1.344 (3)	C11—H11B	0.9700
N4—C6	1.343 (3)	C12—H12A	0.9700
N4—C14	1.457 (3)	C12—H12B	0.9700
N4—C11	1.463 (3)	C13—C14	1.510 (3)
N5—C12	1.461 (3)	C13—H13A	0.9700
N5—C13	1.474 (3)	C13—H13B	0.9700
N5—Mn1^iv^	2.3723 (19)	C14—H14A	0.9700
N5—H5N	0.90 (3)	C14—H14B	0.9700
C1—C2	1.508 (3)		
			
O1—Mn1—O1^i^	180.0	N4—C6—N3	117.5 (2)
O1—Mn1—O3	83.09 (6)	N4—C6—N2	117.0 (2)
O1^i^—Mn1—O3	96.91 (6)	N3—C6—N2	125.4 (2)
O1—Mn1—O3^i^	96.91 (6)	N3—C7—N1	117.7 (2)
O1^i^—Mn1—O3^i^	83.09 (6)	N3—C7—C4	123.4 (2)
O3—Mn1—O3^i^	180.0	N1—C7—C4	118.9 (2)
O1—Mn1—N5^ii^	90.17 (7)	N1—C8—C2	125.8 (3)
O1^i^—Mn1—N5^ii^	89.83 (7)	N1—C8—H8A	117.1
O3—Mn1—N5^ii^	90.74 (7)	C2—C8—H8A	117.1
O3^i^—Mn1—N5^ii^	89.26 (7)	C10—C9—N1	111.2 (5)
O1—Mn1—N5^iii^	89.83 (7)	C10—C9—H9A	109.4
O1^i^—Mn1—N5^iii^	90.17 (7)	N1—C9—H9A	109.4
O3—Mn1—N5^iii^	89.26 (7)	C10—C9—H9B	109.4
O3^i^—Mn1—N5^iii^	90.73 (7)	N1—C9—H9B	109.4
N5^ii^—Mn1—N5^iii^	180.0	H9A—C9—H9B	108.0
C1—O1—Mn1	137.22 (16)	C9—C10—H10A	109.5
C3—O3—Mn1	129.93 (16)	C9—C10—H10B	109.5
C8—N1—C7	118.7 (2)	H10A—C10—H10B	109.5
C8—N1—C9	119.8 (3)	C9—C10—H10C	109.5
C7—N1—C9	121.4 (2)	H10A—C10—H10C	109.5
C5—N2—C6	115.3 (2)	H10B—C10—H10C	109.5
C7—N3—C6	116.3 (2)	N4—C11—C12	110.2 (2)
C6—N4—C14	120.9 (2)	N4—C11—H11A	109.6
C6—N4—C11	122.6 (2)	C12—C11—H11A	109.6
C14—N4—C11	113.1 (2)	N4—C11—H11B	109.6
C12—N5—C13	108.90 (19)	C12—C11—H11B	109.6
C12—N5—Mn1^iv^	116.15 (15)	H11A—C11—H11B	108.1
C13—N5—Mn1^iv^	111.54 (14)	N5—C12—C11	114.3 (2)
C12—N5—H5N	110 (2)	N5—C12—H12A	108.7
C13—N5—H5N	107 (2)	C11—C12—H12A	108.7
Mn1^iv^—N5—H5N	103 (2)	N5—C12—H12B	108.7
O2—C1—O1	123.4 (2)	C11—C12—H12B	108.7
O2—C1—C2	117.6 (2)	H12A—C12—H12B	107.6
O1—C1—C2	118.9 (2)	N5—C13—C14	112.8 (2)
C8—C2—C3	119.0 (2)	N5—C13—H13A	109.0
C8—C2—C1	116.1 (2)	C14—C13—H13A	109.0
C3—C2—C1	124.9 (2)	N5—C13—H13B	109.0
O3—C3—C2	126.0 (2)	C14—C13—H13B	109.0
O3—C3—C4	119.8 (2)	H13A—C13—H13B	107.8
C2—C3—C4	114.3 (2)	N4—C14—C13	111.1 (2)
C7—C4—C5	114.3 (2)	N4—C14—H14A	109.4
C7—C4—C3	123.3 (2)	C13—C14—H14A	109.4
C5—C4—C3	122.4 (2)	N4—C14—H14B	109.4
N2—C5—C4	124.8 (2)	C13—C14—H14B	109.4
N2—C5—H5A	117.6	H14A—C14—H14B	108.0
C4—C5—H5A	117.6		
			
O3—Mn1—O1—C1	−1.4 (3)	C7—N3—C6—N4	175.3 (3)
O3^i^—Mn1—O1—C1	178.6 (3)	C7—N3—C6—N2	−6.2 (4)
N5^ii^—Mn1—O1—C1	−92.1 (3)	C5—N2—C6—N4	−174.7 (3)
N5^iii^—Mn1—O1—C1	87.9 (3)	C5—N2—C6—N3	6.8 (4)
O1—Mn1—O3—C3	1.4 (2)	C6—N3—C7—N1	−177.9 (3)
O1^i^—Mn1—O3—C3	−178.6 (2)	C6—N3—C7—C4	−0.5 (4)
N5^ii^—Mn1—O3—C3	91.5 (2)	C8—N1—C7—N3	177.3 (3)
N5^iii^—Mn1—O3—C3	−88.5 (2)	C9—N1—C7—N3	−2.9 (5)
Mn1—O1—C1—O2	179.3 (2)	C8—N1—C7—C4	−0.1 (5)
Mn1—O1—C1—C2	1.5 (4)	C9—N1—C7—C4	179.7 (3)
O2—C1—C2—C8	−1.0 (4)	C5—C4—C7—N3	5.6 (4)
O1—C1—C2—C8	177.0 (3)	C3—C4—C7—N3	−175.2 (3)
O2—C1—C2—C3	−179.1 (3)	C5—C4—C7—N1	−177.1 (3)
O1—C1—C2—C3	−1.2 (4)	C3—C4—C7—N1	2.1 (4)
Mn1—O3—C3—C2	−1.8 (4)	C7—N1—C8—C2	−1.3 (5)
Mn1—O3—C3—C4	−179.39 (16)	C9—N1—C8—C2	178.8 (4)
C8—C2—C3—O3	−176.6 (3)	C3—C2—C8—N1	0.8 (5)
C1—C2—C3—O3	1.5 (4)	C1—C2—C8—N1	−177.5 (3)
C8—C2—C3—C4	1.1 (4)	C8—N1—C9—C10	92.7 (5)
C1—C2—C3—C4	179.2 (2)	C7—N1—C9—C10	−87.1 (5)
O3—C3—C4—C7	175.3 (2)	C6—N4—C11—C12	−148.9 (3)
C2—C3—C4—C7	−2.5 (4)	C14—N4—C11—C12	51.9 (3)
O3—C3—C4—C5	−5.6 (4)	C13—N5—C12—C11	54.0 (3)
C2—C3—C4—C5	176.6 (3)	Mn1^iv^—N5—C12—C11	−179.1 (2)
C6—N2—C5—C4	−0.7 (5)	N4—C11—C12—N5	−52.9 (4)
C7—C4—C5—N2	−5.0 (4)	C12—N5—C13—C14	−55.0 (3)
C3—C4—C5—N2	175.8 (3)	Mn1^iv^—N5—C13—C14	175.47 (16)
C14—N4—C6—N3	−8.0 (4)	C6—N4—C14—C13	146.2 (3)
C11—N4—C6—N3	−165.6 (3)	C11—N4—C14—C13	−54.2 (3)
C14—N4—C6—N2	173.4 (3)	N5—C13—C14—N4	55.9 (3)
C11—N4—C6—N2	15.8 (4)		

Symmetry codes: (i) −*x*+1, −*y*+1, −*z*+1; (ii) *x*+1, −*y*+3/2, *z*+1/2; (iii) −*x*, *y*−1/2, −*z*+1/2; (iv) −*x*, *y*+1/2, −*z*+1/2.

### Hydrogen-bond geometry (Å, °)

**Table:**

*D*—H···*A*	*D*—H	H···*A*	*D*···*A*	*D*—H···*A*
N5—H5N···O2^v^	0.893 (10)	2.268 (12)	3.149 (3)	169 (3)

Symmetry codes: (v) −*x*+1, *y*+1/2, −*z*+1/2.
